# Thoracic, Peripheral, and Cerebral Volume, Circulatory and Pressure Responses To PEEP During Simulated Hemorrhage in a Pig Model: a Case Study

**DOI:** 10.2478/joeb-2021-0013

**Published:** 2021-12-27

**Authors:** Leslie D. Montgomery, Richard W. Montgomery, Michael Bodo, Richard T. Mahon, Frederick J. Pearce

**Affiliations:** 1 LDM Associates, San Jose, CA, USA; 2 Walter Reed Army Institute of Research, Silver Spring, MD, USA; 3 Current position: Ochsner Medical Center, New Orleans, LA, USA; 4 Naval Medical Research Center, Silver Spring, MD, USA

**Keywords:** Bioimpedance, electrical impedance spectroscopy, systemic arterial pressure, pulmonary arterial pressure, PEEP, bleeding, cerebrovascular reactivity, REG, carotid flow, ICP, pig

## Abstract

Positive end-expiratory pressure (PEEP) is a respiratory/ventilation procedure that is used to maintain or improve breathing in clinical and experimental cases that exhibit impaired lung function. Body fluid shift movement is not monitored during PEEP application in intensive care units (ICU), which would be interesting specifically in hypotensive patients. Brain injured and hypotensive patients are known to have compromised cerebral blood flow (CBF) autoregulation (AR) but currently, there is no non-invasive way to assess the risk of implementing a hypotensive resuscitation strategy and PEEP use in these patients.

The advantage of electrical bioimpedance measurement is that it is noninvasive, continuous, and convenient. Since it has good time resolution, it is ideal for monitoring in intensive care units (ICU). The basis of its future use is to establish physiological correlates. In this study, we demonstrate the use of electrical bioimpedance measurement during bleeding and the use of PEEP in pig measurement.

In an anesthetized pig, we performed multimodal recording on the torso and head involving electrical bioimpedance spectroscopy (EIS), fixed frequency impedance plethysmography (IPG), and bipolar (rheoencephalography – REG) measurements and processed data offline. Challenges (n=16) were PEEP, bleeding, change of SAP, and CO_2_ inhalation. The total measurement time was 4.12 hours.

Systemic circulatory results: Bleeding caused a continuous decrease of SAP, cardiac output (CO), and increase of heart rate, temperature, shock index (SI), vegetative - Kerdo index (KI). Pulse pressure (PP) decreased only after second bleeding which coincided with loss of CBF AR. Pulmonary arterial pressure (PAP) increased during PEEP challenges as a function of time and bleeding.

EIS/IPG results: Body fluid shift change was characterized by EIS-related variables. Electrical Impedance Spectroscopy was used to quantify the intravascular, interstitial, and intracellular volume changes during the application of PEEP and simulated hemorrhage. The intravascular fluid compartment was the primary source of blood during hemorrhage. PEEP produced a large fluid shift out of the intravascular compartment during the first bleeding period and continued to lose more blood following the second and third bleeding. Fixed frequency IPG was used to quantify the circulatory responses of the calf during PEEP and simulated hemorrhage. PEEP reduced the arterial blood flow into the calf and venous outflow from the calf.

Head results: CBF AR was evaluated as a function of SAP change. Before bleeding, and after moderate bleeding, intracranial pressure (ICP), REG, and carotid flow pulse amplitudes (CFa) increased. This change reflected vasodilatation and active CBF AR. After additional hemorrhaging during PEEP, SAP, ICP, REG, CFa signal amplitudes decreased, indicating passive CBF AR. 1) The indicators of active AR status by modalities was the following: REG (n=9, 56 %), CFa (n=7, 44 %), and ICP (n=6, 38 %); 2) CBF reactivity was better for REG than ICP; 3) REG and ICP correlation coefficient were high (R^2^ = 0.81) during CBF AR active status; 4) PRx and REGx reflected active CBF AR status. CBF AR monitoring with REG offers safety for patients by preventing decreased CBF and secondary brain injury.

We used different types of bioimpedance instrumentation to identify physiologic responses in the different parts of the body (that have not been discussed before) and how the peripheral responses ultimately lead to decreased cardiac output and changes in the head. These bioimpedance methods can improve ICU monitoring, increase the adequacy of therapy, and decrease mortality and morbidity.

## Introduction

### PEEP

PEEP is a respiratory/ventilation procedure that is used to maintain or improve breathing in clinical and experimental cases that exhibit impaired lung function. PEEP has been applied to such cases, including, acute respiratory distress syndrome (ARDS) [1], stroke [2], after heart surgery [3], to support breathing of elderly patients [4], and during animal experimentation [5].

The application of PEEP first affects the lungs and pulmonary system. PEEP increases lung volume, alveolar recruitment, and lung compliance thereby reducing the work of breathing [6]. The increase in intrathoracic pressure then depresses the diaphragm extending the effects of PEEP to internal organs [7] and the peripheral circulatory system [8]. PEEP decreases hepatic, splanchnic, and gastric perfusion. PEEP increases systemic circulatory resistance, increases peripheral pooling, and reduces venous return to the heart [9]. Reduced venous return decreases right ventricular preload and increases right ventricular afterload which has the net effect of decreasing the right ventricular stroke volume [10]. In turn, the reduced output from the right ventricle leads to a reduced stroke volume in the left ventricular and a reduction in cardiac output [10]. Decreased cardiac output affects the cerebral circulatory system by increasing intracranial pressure and reducing cerebral perfusion pressure and thereby reducing cerebral blood flow [11].

As stated by [12]: “The effects of mechanical ventilation have been evaluated, but most studies focused on a single variable or circulation regardless of interactions and compensatory mechanisms that should require the simultaneous assessments of multiple circulations.”

Detailed descriptions of the effects of PEEP on individual physiologic systems are given in the Deranged Physiology series [12] of clinical notes. Malbrain [13] gives a description of the impact of intra-abdominal pressure on end-organ function and shows the interaction between multiple physiologic systems.

Shekerdermian and Bohn [8] have proposed a simplified three-compartment model of the cardiovascular effects of mechanical ventilation. However, the above articles do not provide a detailed description of the peripheral [calf] circulation or the intracranial responses to PEEP.

### Hemorrhage

Hemorrhage is a leading cause of death in both civilian and military trauma [15, 16, 17]. In spite of significant advancements in the pathophysiology of hemorrhagic shock [HS] and its treatment, the mortality rate remains high [17]. Extensive bleeding due to unintentional injury remains the principal cause of death for US civilians [18]. Hemorrhagic shock remains a leading cause of morbidity and mortality from battlefield injuries [18]. In a rat study, it was documented, that brain injury together with hemorrhagic shock causes persistent impairment of CBF and brain tissue oxygen tension, increasing the probability of cortical spreading depolarizations that likely contribute to secondary neuropathology and compromise neurological recovery [19].

Continuous monitoring of hemodynamic, autonomic, and/or metabolic responses may provide earlier recognition of hemorrhage than standard vital signs and allow interventions before the onset of hypovolemic shock [20]. The Shock index (SI) is the ratio of heart rate (HR) to systolic arterial pressure (SAP) and may be more useful in early hemorrhage than either vital sign alone [21]. The vegetative balance - Kerdo index [22] was useful in the quantification of cardiovascular stress in hemorrhagic shock [23].

### EIS

Hemorrhage and PEEP is not only a CBF manipulation but also triggers a change in body water compartments (intra-and extra-cellular and vascular fluid shift) which can be measured by BIS non-invasively. The EIS (Z-Scan-2, U.F.I. Inc, Morro Bay, CA) used in this study, combines a fixed frequency Impedance Plethysmograph [IPG] and a multi-frequency electrical impedance spectrograph (EIS) into one unit. The IPG mode was used to quantify the total segmental conductive volume and associated circulatory parameters [24]. The EIS mode was used to monitor segmental intracellular and extracellular compartment volumes as is done by other EIS devices. However, our proprietary software was then used to divide the extracellular compartment volume into its intravascular and interstitial components. The electrical bioimpedance spectrographic mode was validated [25] and used [26, 27] to monitor fluid shifts between the intracellular, interstitial, and intravascular compartments during dialysis. This instrumentation was used in this study to monitor relative changes in interstitial and intravascular compartment volumes during simulated hemorrhage and the use of PEEP [28].

### REG

Early bioimpedance research began with thoracic [cardiac] measurements, which were soon followed by measurements of brain circulation. The term “rheoencephalography” was first used by Jenkner to refer to this technique and more recently the Food and Drug Administration definition has been stated that “A rheoencephalograph is a device used to estimate a patient’s cerebral circulation by electrical impedance methods with direct electrical connections to the scalp or neck area” [29]. The original REG devices were a four-electrode system, later modified to two electrodes. The electrical impedance method (measuring blood flow by alternating current) is known in clinical practice however, it is used mostly in cardiology to measure cardiac output and peripheral circulation (summative blood flow in the limbs). REG is based on monitoring pulse synchronous variations in cranial electrical impedance over time. REG pulse wave amplitude is due to the conductivity differences between brain tissue, and cerebrospinal fluid and blood, with blood and cerebrospinal fluid being better conductors than the brain and other “dry” tissue. The significant physiological information derived from the REG signal relates to vasoconstriction and vasodilation in the brain. This is manifested by decreasing and increasing REG, amplitudes, respectively. The early publication described that REG can be used to detect brain arteriosclerosis and elevated ICP [30]. The units of these amplitude changes are measured in Ohms, however, there are no normative values associated with the REG amplitude values due to the many factors that can affect it [31].

Various correlations have been established between REG and CBF (volume, flow, or pressure, detailed by Jenkner [32]). The consensus from a review of published REG literature is that the REG signal represents volume change. REG pulse amplitude is quantified most frequently using its derivative or integral [33, 34]. Both variables are sensitive to changes in CBF induced by perturbations such as CO_2_ inhalation, Trendelenburg position, carotid occlusion, and hemorrhage. The REG pulse amplitude relationship to cerebral blood volume was previously documented in humans with the radio-iodinated human serum albumin method during CO_2_ inhalation [35]. As a result of this study, it was determined that the REG pulse wave amplitude increased reflecting the intracranial volume increase. In vitro study clearly confirmed this statement [36]. Earlier studies [31, 32] made an initial effort to establish pathophysiological correlates to REG [30, 34] but did not mention CBF AR. Previous studies documented that REG reflects cerebrovascular reactivity [40], has a correlation to ICP [35, 38], laser Doppler flow [39], carotid flow [40] and can be used for patient monitoring instead of ICP [42]. It was documented that REG indicated cerebral vasospasm before systemic reaction caused by complement activation/cytokine storm in pigs [42]. We described multimodal CBF AR changes during hemorrhage and various SAP changes in pigs [43].

Considering the above, the goals of this descriptive case study were:

1. to use extensive invasive and noninvasive techniques to provide multiple system responses to PEEP, including the calf in intracranial regions,
2. to demonstrate how the use of different types of bioimpedance instruments [bipolar implanted electrodes for rheoencephalography, fixed frequency impedance plethysmography, and multi-frequency impedance spectroscopy can give additional information during clinical and experimental studies, and
3. to define primary parameters that can be used to assess the responses of various physiologic systems to the application of PEEP.

## Materials and Methods

Following surgical implantation of catheters and sensors, under anesthesia, the following CBF AR challenges were used: hemorrhage, PEEP, CO_2_ inhalation, and transitory SAP change during switching isoflurane anesthesia to propofol (n=16 challenges). After measurements, the pig was euthanized by lethal hemorrhage. The pig (68.7 kg) was anesthetized with isoflurane and propofol/ketamine anesthesia and spontaneously breathing. Details were described previously [43].

### Simulated Hemorrhage

**Figure 1:**
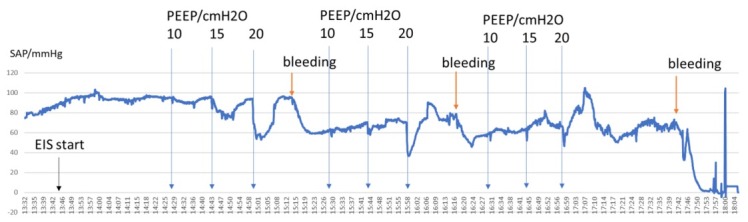
Mean arterial pressure (SAP) during the recoding and challenges (bleeding and PEEP). Y-axis is in mmHg; X-axis is clock time. EIS recording stated at 13.43.

The amount of blood withdrawn during the simulated hemorrhage test is shown in Figure 1, The baseline period [0- 90 min] is followed sequentially by three bleedings: each at elapsed times of 80, 150, and 210 min. Bleedings were performed in three steps, each time 15 % of the estimated blood volume (0.669 L) was withdrawn; after the second bleeding it was 1.339 L (30 %), and the third time total removed volume was 2.009 L (45%).

### Application of PEEP

Sequential levels of PEEP were administered three times during the experiment to counteract the physiologic effects of the simulated hemorrhages. Each series of PEEP consisted of increased levels of the applied pressure of 10, 15, and 20 cm H_2_O. The first series of PEEP took place before the first bleeding. The second and third PEEP took place at the time of initiation of the first and second bleed periods. A ventilator (Invent 201, VersaMed, Pearl River, NY) generated PEEP with 10, 15, and 20 cm H_2_O pressures lasting about 8 minutes three times, separated with breaks.

### Data processing

EIS data was stored on a laptop with a 100 Hz sampling rate. Physiological signals were sampled with a 200 Hz analog-digital conversion rate using DASH-18 (Astro-Med, West Warwick, RI). The total recorded time was 4.12 hours in 3 files. Serial (and analog) data were collected with DREW (Army Institute of Surgical Research, San Antonio, TX) with a 12/min sampling rate. Analog signals were processed offline with DataLyser, an in-house developed program. DataLyser is based upon LabWindows/CVI program (National Instruments, Austin, TX) specifically developed to display, store, and quantify analog physiological signals.

REG integral was calculated after creating the first derivative and turning negative numbers into positive. Running integral calculation was used to smooth the trace with 60-sec windows. PRx and REGx calculations were made identically as was given in the ICM+ program [44, 45]. In case REG was used instead of ICP it was called REGx [45]. Data processing involved 1) visual evaluation of CBF AR responses during SAP changes; 2) automated calculation of CBF AR status; 3) comparing REG integral and ICP mean values during 15 and 20 cm H_2_O PEEP; 4) status of systemic circulation was compared by student t-test, involving 30 minutes data at the start and the end of the recording. SAP and CO were compared by their correlation coefficient involving time window from 14.00 till 17.40, 5) pulse pressure (PP) was calculated as systolic-diastolic blood pressure; 6) calculation of shock index (SI: Heart rate in beats per minute/Systolic blood pressure) [46] and Kerdo index - KI: (1 − diastolic blood pressure/heart rate) + 100 [23] modified by Sipos [47] was calculated covering 4.12 hours recording time. Statistical analysis was performed in Excel (Microsoft, Redmond, WA). Probability was considered significant at P <0.05.

### Ethical approval

The research related to animals use has been complied with all the relevant national regulations and institutional policies for the care and use of animals.

## Results

The results of this study are presented in two sections below. The first part will give the results of the invasive and non-invasive instrumentation that was used to quantify the physiologic responses of the various body systems to the application of PEEP during simulated hemorrhage. The second part will present parameters and displays that can be used by physicians at the bedside to monitor the effects of PEEP during treatment procedures.

Results are presented in the sequence of responses in various bodily systems as described in the introduction. The results are presented that are most directly affected by the administration of PEEP and are related to the hypothesis that PEEP reduces venous return to the heart which in turn leads to reduced cardiac output.

Mean pulmonary arterial pressure (PA-M) is shown in the figures below to provide consistent insight into the various parameter value changes during the simulated hemorrhage periods.

### Physiologic responses to PEEP

Pulmonary Circulation

Systemic (calf) circulation

**EIS-related results**

The impedance spectroscope used in this investigation first quantifies the intracellular and extracellular resistance of the calf. These resistance values are then used to calculate the individual compartment volumes which are then plotted in Figure 3, normalized to the mean of the values during the period before the first bleeding.

**Figure 2:**
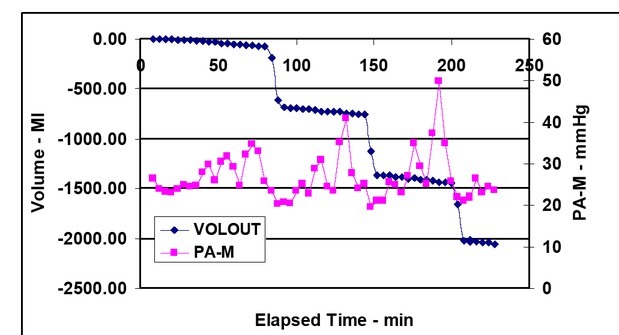
Mean pulmonary arterial pressure (PA-M) and simulated hemorrhage (VOLOUT) vs elapsed time. PA-M is not affected by the amount of bleeding prior to the first bleeding. However, PEEP produces an increase in PA-M following the first and second bleed periods.

**Figure 3:**
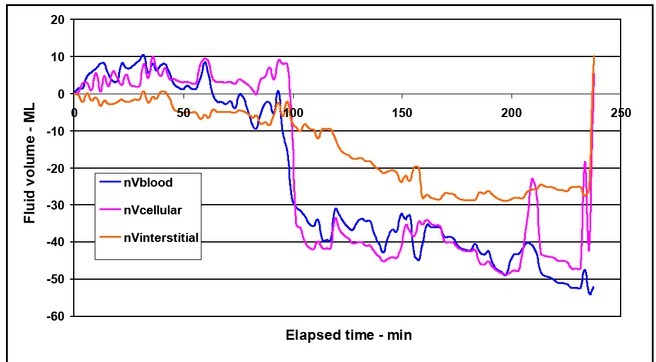
Normalized intravascular (nVblood), cellular (nVcellular) and interstitial (nVinterstital) calf compartment volumes vs. elapsed time.

**Figure 4:**
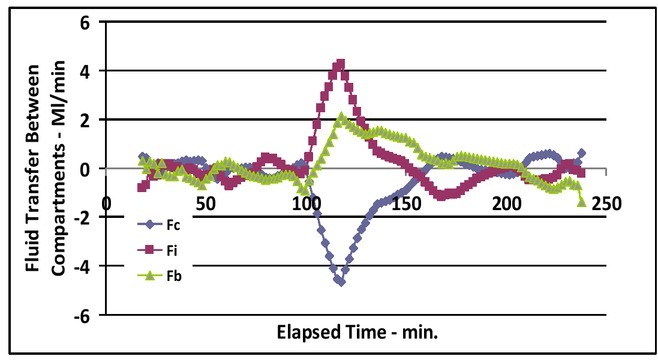
Fluid transfer in and out of the cells (Fc), interstitial space (Fi), and intravascular compartment (Fb) vs. elapsed time.

Positive values indicate fluid transfer out of each compartment and negative values indicate fluid transfer into each compartment. No fluid transfer took place between any of the three fluid compartments prior to the first bleeding. A large amount of fluid was transferred from the interstitial and intracellular compartments into the intravascular space following the first bleeding. Smaller amounts of fluid were transferred between the three compartments after the first bleed period (Figure 4).

**IPG-related results**

**Figure 5:**
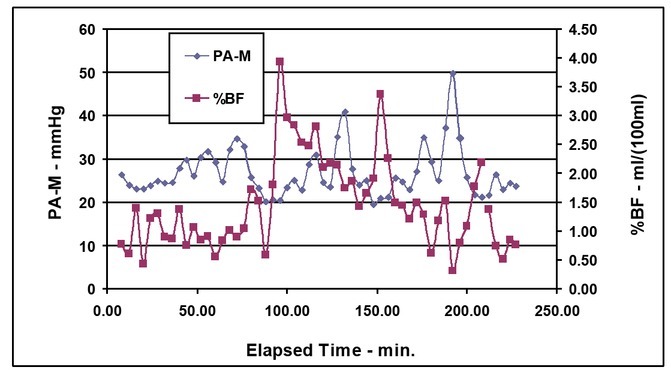
Mean pulmonary arterial pressure (PA-M) and calf percent blood flow (%BF) vs. elapsed time.

Note that calf blood flow is not affected by PA-M before the first bleeding. PA-M does cause an increase in %BF following the first sequence of PA-M and then %BF decreases during subsequent bleed periods. PA-M does produce an increase in %BF during the second and third bleed periods (Figure 5).

**Figure 6:**
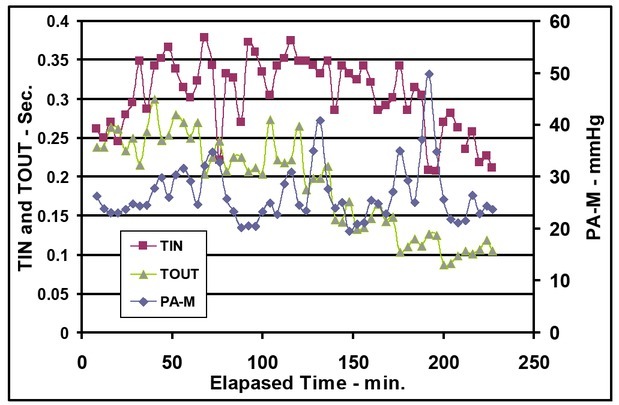
Mean pulmonary arterial pressure [PA-M], time of arterial blood inflow (TIN), and time of venous outflow (TOUT) vs. elapsed time.

Note that both TIN and TOUT increase before the first bleeding. TIN remains relatively constant during the first and second bleed periods while TOUT decreases following the first bleeding and continues to decrease throughout the rest of the experimental duration (Figure 6).

Torso Circulation

**Figure 7:**
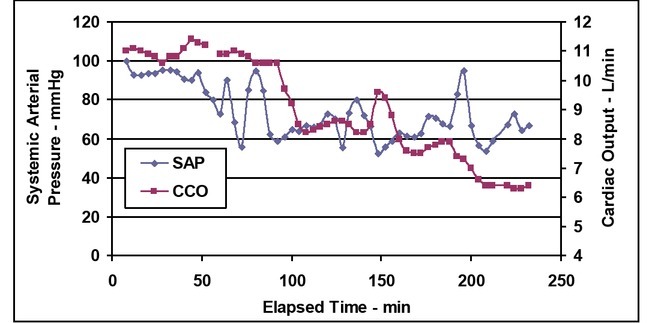
Systemic arterial pressure (SAP) and continuous cardiac output (CCO) vs. elapsed time.

The CCO values were multiplied by 100 so they would fit the same range of the linear Y-axis CO numbers. The SAP and CO decreased during the time of recording because of bleedings. Their correlation was 0.58, The difference was statistically significant between the start and end of SAP P=0.0005, for CO was P<0.0001 (Figure 1.) During the recording time, PP and PAP change invertedly: PP decreased, while PAP increased as a function of bleeding (Figure 2). Their correlation was low during the total recoded time (R^2^ 0.003) but increased after 2^nd^ bleeding (R^2^ 0.511). The difference was statistically not significant (P=0.236) between the start and end for PAP but significant for PP (P=0.0003) (Figure 7).

**Figure 8:**
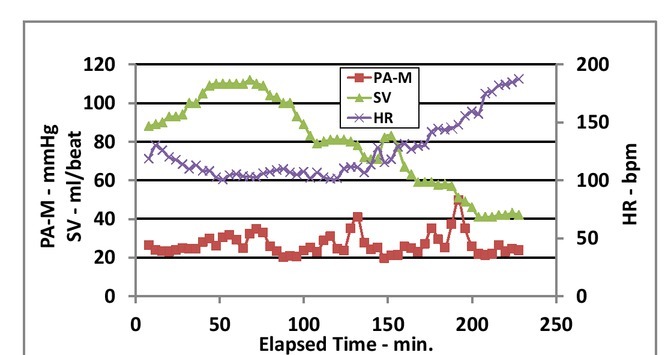
Mean pulmonary arterial pressure (PA-M), stroke volume (SV), and heart rate (HR) vs. elapsed time.

Cardiac output is the product of stroke volume (SV) and heart rate (HR). Heart rate increases following the first bleeding. However, this increase was found to be a result of increased internal temperature of the pig, perhaps due to the effect of the anesthesia used (Figure 8).

**Figure 9:**
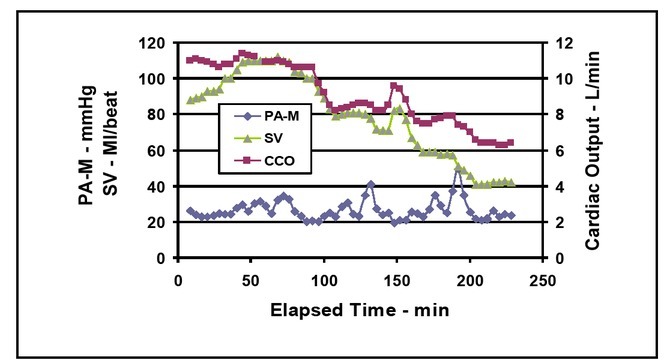
Mean pulmonary arterial pressure (PA-M), stroke volume (SV), and mean cardiac output (CCO) vs. elapsed time.

Stroke volume changes were the predominant factor affecting the cardiac output (CCO). Stroke volume was therefore used to represent the effect of PEEP on the heart during simulated hemorrhage (Figure 9).

**Figure 10:**
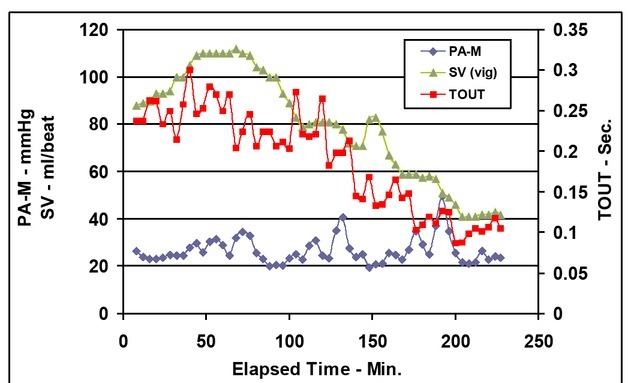
Mean pulmonary arterial pressure (PA-M), stroke volume (SV), and time of venous outflow (TOUT) vs. elapsed time.

Figure 10 shows the two main variables that lead to the decrease in cardiac output during simulated hemorrhage. Venous return from the systemic circulation (TOUT) decreases which then leads to a reduced stroke volume (SV) and subsequently causes a decrease in cardiac output.

### Cerebral Circulation – REG-related results

Before bleeding, the decrease in SAP caused by PEEP administration was associated with increases in the amplitudes of the REG, CF, and ICP signals showed vasodilatation, which reflects active CBF AR. CBF and LDF signals often passively followed SAP. After hemorrhaging during PEEP, the signal amplitudes registered for REG, CF, and ICP showed corresponding decreases in SAP, indicating decreased CBF, which reflects passive CBF AR. The sequence of active AR was led by REG (n=9, 56 %), followed by CFa (n=7, 44 %) and ICP (n=6, 38 %).

CBF AR parameters are given in Table 1, CBF AR status was calculated as the relationship to SAP change: identical change of modality CBF AR was considered as passive (-); in the case of inverted phase, CBF AR was considered as active (+). Artifact contaminated signals, mixed change (+/-), or lack of change (0) were excluded from evaluation. Legend: SAP: systemic arterial pressure; CFa: carotid flow pulse amplitude; mean: CFm: carotid flow mean; REG1d: REG first derivative; CBF: absolute blood flow; LDF: laser Doppler flow; ICP: intracranial pressure; CVP: central venous pressure; PAP: pulmonary arterial pressure; pO_2_. pulse oximetry pulse amplitude; CO_2_. exhaled CO_2_ concentration; +: increase - i.e. identical change; -: decrease - i.e. opposite phase; 0. no data or artifact, no change; iso-prop transit: transition of anesthesia from isoflurane to propofol.

**Table 1:** Summary of CBF AR changes.

Challenge	SAP	CFa	CFm	REG1d	CBF	LDF	ICP	CVP	PAP	CO2	pO2
iso-prop											
transit	+	-	-	-	-	+	-	-	+/-	0	-
PEEP 10	-	+	-	+	+	-	+	-	0	+	-
PEEP 15	-	+	-	+	+/-	+	+	-	0	+	-
PEEP 20	-	+	-	+	+/-	+	+	-	-	+	-
hemorrhage 1	-	+	-	+	+	+	-/+	+	+	-	-/+
PEEP 10	0	0	-/+	+	0	0	+	-	-	+	-
PEEP 15	-	-/+	-/+	+	+	0	+	-	-	+	-
PEEP 20	-	-/+	-/+	+	-/+	-/+	+	-	+	+	-
CO_2_ inhalation	+	0	0	0	+	0	+	0	-	+	-
hemorrhage 2	-	+	+/-	+	-	0	-	+	+	0	+/0
PEEP 10	0	0	+	-	+	0	+	-	-	+	-
PEEP 15	+	-	+	-	+	0	+	+	-	+	-
PEEP 20	-/+	-	-	-	+	+	+	+	-	+	-
hemorrhage 3	-	+	-	+	+	0	-	-	-	+	0
PEEP 10	+	0	0	0	0	0	+	+/-	+	+	+
lethal											
bleeding	-	+/-	-	+/-	-	-	-	-/+	+/-	-/+	+/-

**CBF AR active**		**7**	**1**	**9**	**4**	**3**	**6**	**3**	**4**	**5**	**3**

**Figure 11:**
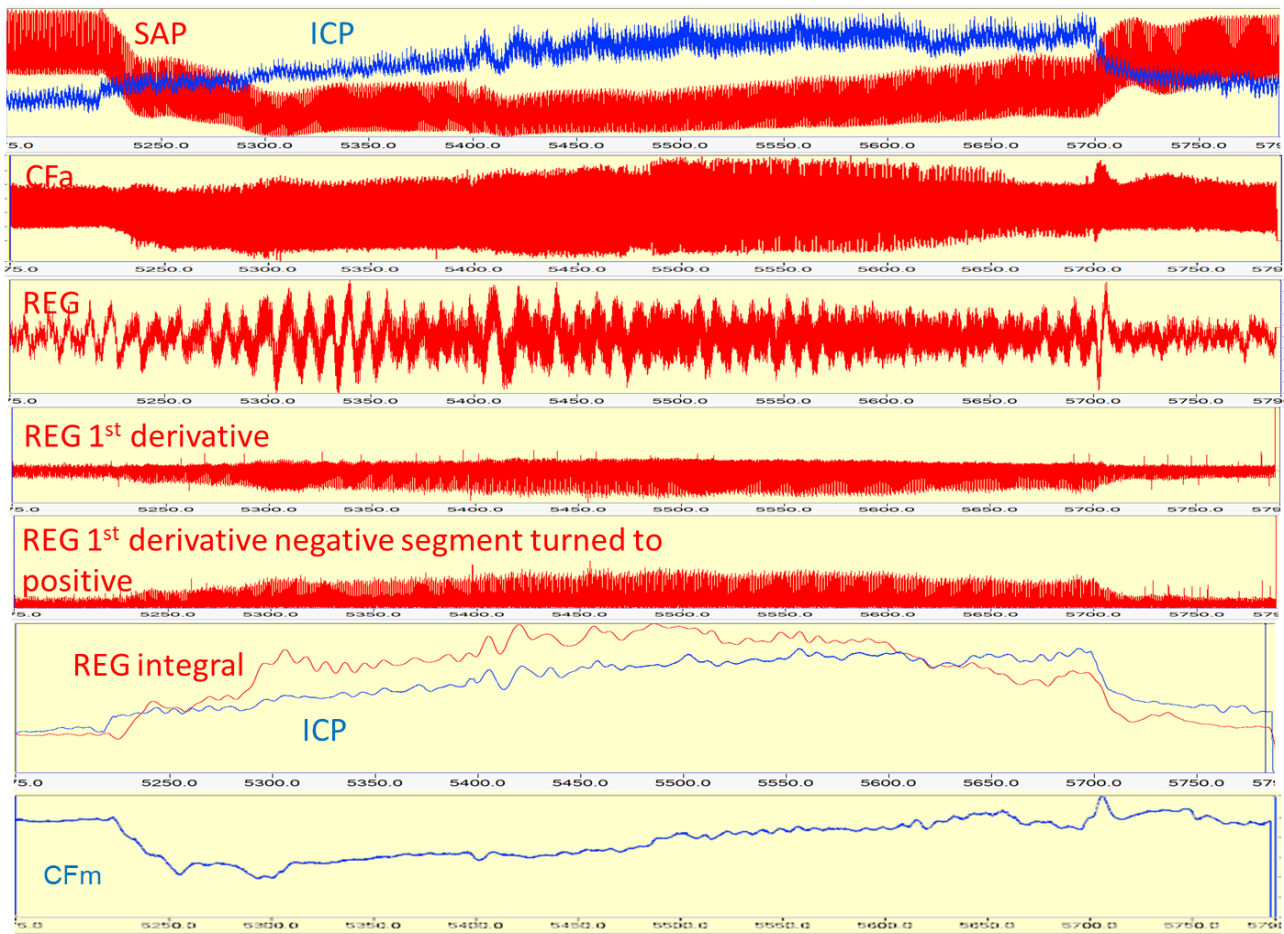
Continuous analog parameters (as a strip chart) vs. time. CBF AR is active.

Twenty cm H_2_O PEEP caused SAP decrease which triggered an increase of ICP, carotid flow (CFa), and REG pulse amplitudes – Figure 11, The first step of REG data processing was creating the 1^st^ derivative of REG pulse amplitude which eliminated the slow oscillation, caused by respiration The next step was to turn up (+) negative values of the 1st derivative of REG. After it, a running integral was calculated with 60 sec time windows. For the REG integral and ICP values (Table 2), the correlation coefficient was 0.81, Bottom trace: mean carotid flow. NB: CFm does not reflect CBF AR. Pig CBF 9, file 13.32, time window: 51755790.9 (615.94) sec.

**Table 2:** Percent changes of mean ICP and REG integral during 15 and 20 cmH_2_O PEEPs before first (1) and after first bleedings (2), and after second bleeding (3). Values are in percentage of their own baseline. Measurement was made at the highest values. Note that after 2^nd^ bleeding REG values are negative, indicating that CBF AR is passive.

	ICP		REG	
	15 cm H_2_O	20 cm H_2_O	15 cm H_2_O	20 cm H_2_O
1	132	148	15	31
2	133	148	28	40
3	74	48	-19	-41

Hemorrhage elicited 1) a decrease in SAP and transitory increases in ICP, REG, and CF amplitude; 2) PEEP resulted in a decrease in SAP and increases in ICP, REG, and CF amplitude; 3) PEEP after hemorrhage caused decreases in SAP, ICP, REG, and CF amplitudes. When CBF AR was present, it was detected by an increase of ICP, REG, and CFa. Following severe hemorrhage, CBF AR was lost; ICP, REG, and CFa passively followed SAP decrease.

### CBF AR test

Effect of 20 cm H20 PEEP, after 2nd hemorrhage

**Figure 12:**
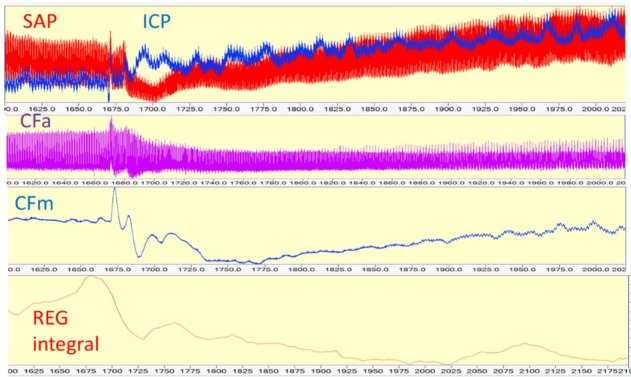
Continuous analog parameters (plotted as a strip chart) vs. time. CBF AR is passive.

Blood loss of about 1.3 L with an estimated shed blood volume of 30%. Hemodynamic status: SAP 90/67, mean 75 mmHg. CBF AR impaired. ICP passively follows SAP increase, except transient active AR at the start of SAP decrease, during 50 sec. SAP decrease elicited a decrease in CFa and REG integral. Pig CBF 9, file 16.30, time window: 1600-2200 (600) sec (Figure 12).

**Figure 13:**
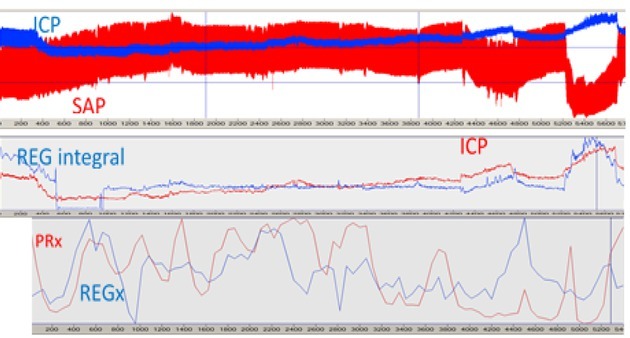
Intracranial pressure (ICP), systemic arterial pressure (SAP), REG integral (REG integral), PRx, and REGx plotted as analog strip chart vs. time. The first number of PRx and REGx calculations starts at 300 sec of the original signal. Pig CBF 9, file 13:32, time window 96.52 min.

Two types of CBF AR calculation before and during PEEP are shown in Figure 13 *Upper trace*: on the left side, SAP increased and ICP decreased because of isoflurane/ propofol anesthesia alteration and the start of ketamine administration (120mL/h). SAP red, REG blue, On the right side, there are two SAP decreases, caused by 15 and 20 cm H_2_O PEEP as well as ICP elevation. Before they were a 10 cm H_2_O PEEP as well but it did not change SAP. *Middle trace*: REG integral and mean ICP, Both indicate an increase, corresponding to the SAP decrease. *Lower trace*: PRx and REGx, tracking CBF AR in real-time. On the left side, both are decreasing, trending to -1, indicating active CBF AR. Similarly on the right side when PEEP challenges started. The first number of PRx and REGx calculations starts at 300 sec of the original signal. Pig CBF 9, file 13:32, time window 96.52 min.

## Discussion

In this descriptive study, we demonstrated the use of three types of bioimpedance methods by measuring torso, peripheral, and brain circulation, noninvasively and correlated to invasive measures. Related results of this pig group are detailed previously [43, 48]. Recent results indicate that 1) EIS reflects body fluid shifts; 2) IPG quantified the circulatory responses of the calf; 3) REG accurately reflects cerebrovascular responsiveness (CBF AR) similarly to ICP and carotid flow pulse amplitude. The clinical importance of our findings was pointed out previously: The potential for PEEP to evoke neurologic complications in patients who have an intracranial disease and that the presence of the pulmonary disease may attenuate these deleterious side effects [50].

### PEEP

Increased intrathoracic pressure due to PEEP has the potential for reducing SAP. Such change could critically reduce cerebral perfusion pressure [CPP] CPP=SAP-ICP [49]. PEEP will be expected to decrease cardiac output mostly by decreasing venous return and right ventricular stroke volume. Hence PEEP and hypovolemia would tend to amplify the decrement in CO. However, we have a venous capacitance (as high as 25% blood volume) that can be called upon and other compensatory mechanisms and could mask some effects of true hypovolemia (especially as it relates to blood pressure). With that in mind, the exact relationship between PEEP and CO is variable. Of note, there does seem to be a level (maybe around a PEEP of 20) where a "waterfall effect'' can occur with dramatic falls in CO and blood pressure. Part of the variable effect of PEEP is related to regional pericardial pressure/regional pleural pressures and lung compliance.

PEEP is applied during the end of expiration to maintain the alveolar pressure above atmospheric pressure. PEEP is different from continuous positive airway pressure (CPAP) because this one refers to a positive pressure maintained during the inspiration and expiration phase of spontaneous ventilation. The benefit of PEEP has been demonstrated in terms of preventing cyclic opening and collapsing alveoli in acute respiratory distress syndrome patients (ARDS). Moreover, protective ventilation, even in noninjury lungs, should be considered such as during the perioperative period aiming to prevent collapsing of alveoli. However, applying PEEP may affect cardiac function and vital organ perfusion by complex mechanisms. To minimize the adverse effects of PEEP in the intensive care unit and in the operating room, better knowledge and understanding of the interaction between heart, lung, and brain during applying PEEP are required [50]. In the 2017 practice guideline, there is no word of PEEP neither cerebral perfusion pressure nor cerebral blood flow [52, 53].

### Clinical aspects

Intensive care unit (ICU) monitoring typically involves invasive (SAP) and noninvasive: electrocardiogram, (ECG) oxygen saturation (pO_2_), temperature, respiration, carbon dioxide (CO_2_) methods. Bedside monitors show these traces and calculate numerical values. A ventilator is a separate device in which several respiratory variables are needed to set up, such as PEEP pressure, volume, frequency, etc. Additionally, in neurocritical care, invasive monitoring is ICP, laser Doppler flow (LDF), brain O_2_/temperature, quantitative CBF; noninvasive monitoring is near-infrared spectroscopy (NIRS), electroencephalogram (EEG), transcranial Doppler (TCD). For details see [54, 55]. ICP is monitored, but not for every patient. It is desired that bioimpedance would be used for brain and cardiorespiratory ICU monitoring since it is continuous, noninvasive, convenient, and cheap.

**Figure 14:**
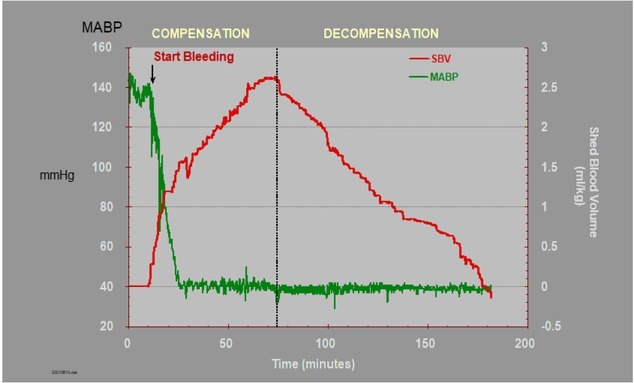
Phases of shock. The shock phases defined as compensatory represent the removal of 50% and 100% of peak shed blood volume (3% of body weight). Shock phases defined as decompensatory represent the return of 20 and 75% of the shed blood volume [58].

During hemorrhage lost blood volume is compensated by interstitial fluid movement into intravascular space while hemorrhagic shock is compensated. Compensation involves an increase in heart rate. After additional blood loss compensation capacity is exhausted and starts a decrease of SAP and after a transient increase of heart rate, it is decreasing (Figure 14). Practical problems were demonstrated previously [43, 57].

A recent guideline [60] states: “We recommend that less invasive devices are used, instead of more invasive devices, only when they have been validated in the context of patients with shock. Best practice”. But searching this document with keywords: “cerebral blood flow” resulted in no-hit! In other words, CBF is not interesting to be monitored in circulatory shock?

However, it was published, that serious hypotension combined with a severe head injury can increase mortality by a factor of four [60]. American Academy of Neurology 2017 Guideline [53]; Neurocritical Care Society Standards [62]; Guidelines [62, 63, 64]; the protocol for the treatment of SARS-CoV-2 [65] does not mention “cerebral blood flow” or “PEEP” but sometimes cerebral perfusion.

Acute respiratory distress syndrome (ARDS) is a life-threatening form of respiratory failure that affects approximately 200 000 patients each year in the United States, resulting in nearly 75 000 deaths annually. Globally, ARDS accounts for 10% of intensive care unit admissions, representing more than 3 million patients with ARDS annually [52].

ARDS exerts a substantial disease burden, with 40% of patients dying in hospitals. Diverse factors, including patient-related factors such as age and illness severity, country-level socioeconomic status, and ventilator management and ICU organizational factors each contribute to the outcome from ARDS. Addressing these issues provides opportunities to improve outcomes in patients with ARDS [64]. Monitoring CBF AR during PEEP is an option to decrease ICU mortality. Probably it would be helpful in the management of severe hypoxemic respiratory failure, too [66]. An experimental study demonstrated, that CBF AR is progressively impaired during septic shock [70].

### Shock and Kerdo index

The SI is the ratio of the HR to SBP. The index is a sensitive indicator of left ventricular dysfunction and can become elevated following a reduction in left ventricular stroke work. The SI can be used in the emergency care and intensive care units to identify patients needing a higher level of care despite vital signs that may not appear strikingly abnormal [68]. Persistent elevation of the SI has been associated with a poor outcome in critically ill patients [69]. The SI is a sensitive indicator of left ventricular dysfunction and displays variability in critical patients displaying normal vital signs [68]. In our current study, the greatest increase in SI was seen after 3^rd^ bleeding. This increase coincided with a heart rate and temperature increase. KI values are indicative of autonomic dysfunction [71]. Kerdo index was modified by Sipos previously [47]. The KI values above 100 indicate sympathicotonia, and values below 100 indicate parasympathicotonia. Both SI and modified KI were increased, SAP and CO were decreased during recording time. PAP reaction during PEEPs increased. PP decrease after 2^nd^ bleeding was identical to loss of CBF AR, indicated by REG. It is a practical experience that pig stress (hemorrhagic shock) reaction involves an increase in body temperature. It is opposite to human and rat reactions. Calculation of shock index and vegetative balance (KI) was useful in quantifying cardiovascular stress [70, 71].

### CBF

PEEP and Cerebral Perfusion Pressure [CPP]. About 20–25% of patients with brain injury developed ARDS, which was associated with high mortality. Guideline for mechanical ventilation in ARDS recommended low tidal volume and moderate to high levels of PEEP. Nevertheless, the use of PEEP in brain injury led to an increase in intrathoracic pressure, impeded venous return, and reduced cerebral venous drainage from superior vena cava. Finally, these effects induced high ICP and CPP. However, in clinical studies, these effects occurred only when applying PEEP more than 15 cm H_2_O in hypovolemic patients [72]. Higher CBF AR response of REG can be explained that REG reflects better the arteriolar blood volume change than the Doppler, which measures a big vessel flow [73]. NB: the organ of CBF AR is the arteriola [74, 75]. A recent MRI study described loss of grey matter in cortical areas directly connected to primary olfactory and gustatory cortex after Covid-19 infection, comped to pre-Covid-19 status [76]. It can be hypothesized that there were CBF -related changes as well, which can be detected with REG much cheaper.

It was demonstrated that BOLD MRI can reflect an early impairment of cerebrovascular reserve after aneurysmal subarachnoid hemorrhage [77]. Since REG reflects CBF AR it is expected that will be able to reflect the same way the impairment of cerebrovascular reserve, tested by CO_2_ inhalation; REG correlated well (AUC= 0.84 with p<0.0001) to laser Doppler flow during CO_2_ inhalation [78].

The clinical importance of these findings is that REG can be measured more conveniently and continuously in humans than Doppler ultrasound. Therefore, the measurement of CBF AR by REG has the potential for use as a life sign monitoring modality in neurocritical care units as well as in the military environment.

### Military relevance

Additionally, to civilian cases, military medicine offers practical use for bioimpedance, since the dominant cause of death is hemorrhage and TBI [15, 79, 80, 81, 82]. Brain-injured patients are known to have compromised CBF AR but currently, there is no non-invasive way to assess the risk of implementing a hypotensive resuscitation strategy in the brain-injured patient [83]. A previous study demonstrated that REG can be used to identify the autoregulatory breakpoint to determine the limit for permissive hypotension. Bioimpedance monitoring can be a help during the transport of wounded service members, independently of the Covid pandemic [84, 85].

In civilian clinical practice, invasive SAP and ICP monitoring are typically used to detect the status of CBF AR. However, in military medical practice, these invasive modalities cannot be used during the transport of injured, deployed military personnel. Moreover, in military medical practice, resuscitation techniques used to treat injured service members on the battlefield can result in the unintended consequence of secondary brain injury. After brain/blast injury cerebral vascular reactivity (CVR) can be lost, which is a bad prognosis. In the absence of the ability to maintain cerebral perfusion, a state of hypotension results in significant ischemia and secondary brain injury. Currently, however, there is no way of assessing the risk of implementing a hypotensive resuscitation strategy in a patient suspected of having traumatic brain injury [60, 87, 89]. If normal CVR is found to be present, then lower blood pressures/cerebral perfusion pressures can be tolerated. Conversely, if CVR is absent, then blood pressures must be maintained at a higher level to prevent cerebral ischemia. A meta-analysis revealed significant benefits of hypotensive resuscitation relative to mortality in traumatic hemorrhagic shock [61]. Damage control resuscitation is the overall guiding concept to emerge from the recent military experience [87]. A Guideline states: “If blood pressure [BP] monitoring is available, maintain target systolic blood pressure 80-90 mmHg” [88].

Traumatic brain injury is a frequent component of the combat casualty's injury profile in addition to hypovolemic hypotension. Furthermore, disruption of CBF AR has been recently associated with poorer outcomes, however, currently; there are no non-invasive approaches to evaluate the status of cerebral autoregulation. A major diagnostic limitation for the blast-induced head-injured patient is the inability to image the cranium using MRI due to the possibility of embedded metal fragments from improvised explosive devices. Additionally, CT angiography sometimes fails to detect vasospasm due to the associated metal artifact. Based on previous results, REG seems to be a practical noninvasive and continuous monitoring modality of traumatic brain and blast injuries, since REG signal seems to be insensitive to the presence of metal fragments [92]. REG monitoring on the battlefield is possible by using a miniaturized REG amplifier [90]. By using additional modalities (ECG, respiration) can help the medic in end-of-life decision making [91, 92]. A study described, that 87.3% of all injury mortality occurred in the pre-medical treatment facility environment [93]. It is also stated, that “To impact the outcome of combat casualties with potentially survivable injury, strategies must be developed to mitigate hemorrhage on the battlefield” Monitoring CBF AR during transport and afterward is one potential tool to decrease combat morbidity and mortality, which caused by secondary brain damage.

## Conclusions

1. We used multiple invasive and noninvasive procedures to present an integrated sequence as to how PEEP affects the different regions/organs of the body – at three levels of PEEP after bleeding
2. Illustrated how different bioimpedance measurements can provide a fuller characterization of physiologic responses to clinical and environmental stress - bipolar to monitor cerebral circulation, the fixed frequency at 50KHz to measure total segment volume and hemodynamic state, impedance spectroscopy to quantify fluid compartment volumes, and fluid transfer between compartments. Lastly, (perhaps) calculation of phase angle and/or cell membrane capacitance to investigate cell hydration.
3. Identified those methods/parameters that can best be used during bedside monitoring and for research.
4. We demonstrated that the use of PEEP in brain-injured and hypotensive patients is safe only if CBF AR is monitored.
5. REG indicated the point at which CBF AR is lost
6. The use of the Shock and Kerdo indices can quantify cardiovascular and autonomic stress responses before vital signs are abnormal.

## Disclaimer

Material has been reviewed by the Walter Reed Army Institute of Research. There is no objection to its presentation and/or publication. The opinions or assertions contained herein are the private views of the author and are not to be construed as official, or as reflecting trueviews of the Department of the Army or the Department of Defense. The research was conducted under an approved animal use protocol in an AAALAC International-accredited facility in compliance with the Animal Welfare Act and all other federal statutes and regulations relating to animals and experiments involving animals and adheres to principles stated in the *Guide for Care and Use of Laboratory Animals*, NRC Publication, 2011 edition. This work was supported by the U.S. Army Medical Research and Materiel Command (D43_0025_2005_WRAIR) and SBIR Grants (1 R43 HL074524-01 and 2 R44 HL074524-02A2).

## References

[1] Huaiwu H, Hu Q, Long Y, Wang X, Zhang R, Su L (2019). Effects of high PEEP and fluid administration on systemic circulation, pulmonary microcirculation, and alveoli in a canine model. J. Appl Physiol.

[2] Georgiadis D, Schwartz S, Baumgartner RW, Veltkamp R, Schwab S (2001). Influence of positive end-expiratory pressure on intracranial pressure and cerebral perfusion pressure in patients with acute stroke. Stroke.

[3] Angerpointner TA, Farnsworth AE, Williams BT (1977). Effects of PEEP on cardiovascular dynamics after open-heart surgery: A new postoperative monitoring technique. The Annals of Thoracic Surgery.

[4] Zhou L, Cai G, Zhihui X, Weng Q, Ye Q, Chen C (2019). High positive end-expiratory pressure levels affect hemodynamics in elderly patients with hypertension admitted to the intensive care unit: a prospective cohort. BMC Pulmonary Medicine.

[5] Beyer J, Beckenlechner P, Messmer K (1982). The influence of PEEP ventilation on organ blood flow and peripheral oxygen delivery. Intensive Care Med.

[6] Deranged Physiology. (2021). Effects of positive pressure ventilation on pulmonary physiology.

[7] Nanas S, Magder S. (1992). Adaptations of peripheral circulation to PEEP. American Review of Respiratory Disease.

[8] Shekerdemian L, Bohn D (1999). Cardiovascular effects of mechanical ventilation. Arch. Dis. Child.

[9] Deranged Physiology (2021). Effects of positive pressure ventilation on cardiovascular physiology.

[10] Boone MD, Jinadasa SP, Muller A, Shaefi S, Kasper EM, Hanafy KA (2017). The effect of positive end-expiratory pressure on intracranial pressure and cerebral hemodynamics. Neurocrit. Care.

[11] Lefrant JY, Juan JM, Bruelle P, Demaria R, Cohendy R, Aya G (2002). Regional blood flows are affected differently by PEEP when the abdomen is open or closed: an experimental rabbit model. Can J Anesth.

[12] Deranged Physiology (2021) Haemodynamic changes during the mechanical breath.

[13] Malbrain M (2009). Abdominal compartment syndrome. Medicine Reports.

[14] Alam HB (2010;9). Advances in Resuscitation Strategies. Int J Surg.

[15] Bellamy R, Safar P, Tisherman SA (1996;24). Suspended Animation for Delayed Resuscitation. Crit Care Med.

[16] Bouglé A, Harrois A, Duranteau J (2013). Resuscitative strategies in traumatic hemorrhagic shock. Ann Intensive Care.

[17] (2009). Leading Causes of Death by Age Group, United States -.

[18] Geeraedts L, Kaasjager H, van Vugt A (2009;40). Exsanguination in trauma: A review of diagnostics and treatment options. Injury.

[19] Leung LY, Wei G, Shear DA, Tortella FC (2013). The acute effects of hemorrhagic shock on cerebral blood flow, brain tissue oxygen tension and spreading depolarization following penetrating ballistic-like brain injury. J Neurotrauma.

[20] Convertino V, Ryan K, Rickards C (2008;64). Physiological and medical monitoring for en route care of combat casualties. J Trauma.

[21] Rady MY, Nightingale P, Little RA, Edwards JD (1992;23). Shock index: A reevaluation in acute circulatory failure. Resuscitation.

[22] Kerdo I (1966;29). An index for the evaluation of vegetative tonus calculated from the data of blood circulation. Acta Neuroveg (Wien).

[23] Bodo M, Bothwell S, Dorsey J (2010;48). Comparison of Circulatory Stress Indicators in a Swine Model. Kalokagathia.

[24] Montgomery LD, Dietrich MS, Armer JM, Stewart BR, Ridner SH (2012). Segmental blood flow and hemodynamic state of lymphedematous and nonlymphedematous arms. Lymphatic Research and Biology.

[25] Montgomery LD, Gerth WA, Montgomery RW, Lew SQ, Klein MD, Stewart JM (2013). Monitoring intracellular, interstitial, and intravascular volume changes during fluid management procedures. Med. Biol. Eng. Comput.

[26] Montgomery LD, Montgomery RW, Gerth WA, Lew SQ, Klein MD, Stewart JM (2017). Bioimpedance monitoring of cellular hydration during hemodialysis therapy. Hemo. Int.

[27] Montgomery LD, Montgomery RW, Gerth WA, Laughry M, Lew SQ, Velasquez MT (2017). A system to monitor segmental intracellular, interstitial, and intravascular volume and circulatory changes during acute hemodialysis. J. Electr. Bioimp.

[28] Montgomery L., Montgomery R., Gerth W., Bodo M., Stewart J., Loughry M (2019). Segmental intracellular, interstitial, and intravascular volume changes during simulated hemorrhage and resuscitation: A case study. Journal of Electrical Bioimpedance.

[29] (1997). Rheoencephalograph (a) Identification Code of Federal Regulations Title 21, vol 8, Sec 882.1825, Washington DC: US Government Printing Office; Revised as of April 1.

[30] McHenry LC. (1965). Rheoencephalography: a clinical appraisal. Neurology.

[31] Moskalenko Yu (1980). Ye (ed). Biophysical aspects of cerebral circulation. Oxford, Pergamon.

[32] Jenkner FL (1986). Clinical rheoencephalography: A noninvasive method for automatic evaluation of cerebral hemodynamics. Ertldruck, Vienna, A.

[33] Jacquy J, Dekonick WJ, Piraux A, Calay R, Bacq J, Levy D, Noel G (1974). Cerebral blood flow and quantitative rheoencephalography. Electroenceph Clin. Neurophysiol.

[34] (1968). Hadjiev D: A new method for quantitative evaluation of cerebral blood flow by rheoencephalography. Brain Res.

[35] Bodo M, Racz J, Ilias L, Pasztor A, Vajda J, Weinstein GB, Pasztor E, Moskalenko YE (1986). Rheoencephalographic changes during increased intracranial pressure. In: Krieglstein J. (ed): Pharmacology of cerebral ischemia. Elsevier, Amsterdam, 265269.

[36] Bodo M, Garcia A, Pearce F, van Albert S, Armonda R (2010). Influence of volume and flow change on the electrical impedance signal (in vitro). J. Phys.: Conf. Ser.

[37] Bodo M (2010). Studies in rheoencephalography (REG). J Electr Bioimp.

[38] Bodo M, Simovic M, Pearce F, Ahmed A, Armonda R (2015). Correlation of rheoencephalogram and intracranial pressure: results of a rat study. Physiol. Meas.

[39] Bodo M, Sheppard R, Hall A, Baruch M, Laird M, Tirumala S, Mahon R (2016). Correlation of rheoencephalography and laser Doppler flow: a rat study. J Electr Bioimp.

[40] Bodo M, Pearce F, Van Albert S, Armonda R (2007). Rheoencephalogram reflects cerebral blood flow autoregulation in pigs. In: Scharfetter H., Merva R. (Eds). ICEBI.

[41] Bodo M, Rahaman V, Cannizzaro L, Iwuchukwu I, Hirzallah M (2021). Non-invasive brain monitoring with electrical bioimpedance using rheoencephalography for the evaluation of cerebral blood flow autoregulation and intracranial pressure. Military Health System Research Symposium, Kissimmee, FL.

[42] Bodo M, Szebeni J, Baranyi J, Savay S, Pearce FJ, Alving CR, Bünger R (2005). Cerebrovascular involvement in liposome - induced cardiopulmonary distress in pigs. J Liposome Res.

[43] Bodo M, Montgomery LD, Pearce FJ, Armonda R (2018). Measurement of cerebral blood flow autoregulation with rheoencephalography: a comparative pig study. J Electr Bioimp.

[44] ICM+ program: Software for Brain Monitoring in Neurological Intensive Care.

[45] Brady KM, Mytar JO, Kibler KK, Easley RB, Koehler RC, Czosnyka M, Smielewski P, Zweifel C, Bodo M, Pearce FJ, Armonda RA Monitoring cerebrovascular pressure reactivity with rheoencephalography. 2010 J. Phys.: Conf. Ser.

[46] Rady MY, Nightingale P, Little RA, Edwards JD (1992;23). Shock index: A re¬evaluation in acute circulatory failure. Resuscitation.

[47] Bodo M, Thuroczy G (1995). A complex cerebrovascular screening system (Cerberus). Medical Progress through Technology.

[48] Montgomery LD, Montgomery RW, Gerth WA, Bodo M, Stewart JM, Loughry M (2019). Segmental intracellular, interstitial, and intravascular volume changes during simulated hemorrhage and resuscitation: A case study. J Electr Bioimp.

[49] Aidinis SJ, Lafferty J, Shapiro HM (1976). Intracranial responses to PEEP. Anesthesiology.

[50] Vargas M, Sutherasan Y, Gregoretti C, Pelosi P (2014). PEEP role in ICU and operating room: from pathophysiology to clinical practice. Scientific World Journal.

[51] Geocadin RG, Wijdicks E (2017). Practice guideline summary: Reducing brain injury following cardiopulmonary resuscitation. Report of the Guideline Development, Dissemination, and Implementation Subcommittee of the American Academy of Neurology. Neurology.

[52] Fan E, Del Sorbo L (2017). American Thoracic Society, European Society of Intensive Care Medicine, and Society of Critical Care Medicine. An Official American Thoracic Society/European Society of Intensive Care Medicine/Society of Critical Care Medicine Clinical Practice Guideline: Mechanical Ventilation in Adult Patients with Acute Respiratory Distress Syndrome. Am J Respir Crit Care Med.

[53] Le Roux P, Menon DK (2014). Consensus summary statement of the International Multidisciplinary Consensus Conference on Multimodality Monitoring in Neurocritical Care: a statement for healthcare professionals from the Neurocritical Care Society and the European Society of Intensive Care Medicine. Neurocrit Care.

[54] Czosnyika M, Hutchinson P, Kirkpatrick P J, Pickard J D (2009). Monitoring of the Brain: Pressures, flows, and brain tissue probes. In: Jallo J, Loftus CM. editors. Neurotrauma and Critical Care of the Brain, Thieme, New York.

[55] Damhorst GL, Tyburski EA, Brand O, Martin GS, Lam WA (2019). Diagnosis of acute serious illness: the role of point-of-care technologies. Curr Opin Biomed Eng.

[56] Bodo M, Pearce FJ, Baranyi L, Armonda R (2005). Changes in cerebral blood flow modalities during hemorrhage in rats. ATACCC Conference August 15-17.

[57] Cecconi M, De Backer D, Antonelli M, Beale R, Bakker J, Hofer C, Jaeschke R, Mebazaa A, Pinsky MR, Teboul JL, Vincent JL, Rhodes A (2014). Consensus on circulatory shock and hemodynamic monitoring. A task force of the European Society of Intensive Care Medicine. Intensive Care Med.

[58] Manley G, Knudson MM, Morabito D, Damron S, Erickson V, Pitts L (2001). Hypotension, hypoxia, and head injury: frequency, duration, and consequence Arch Surg.

[59] Moheet AM, Sarah L (2018). Livesay SL et al. Standards for Neurologic Critical Care Units: A Statement for Healthcare Professionals from The Neurocritical Care Society. Neurocrit Care.

[60] Cook AM, Jones GM (2020). Guidelines for the Acute Treatment of Cerebral Edema in Neurocritical Care Patients. Neurocrit Care.

[61] Torbey MT, Bosel J (2015). Evidence-Based Guidelines for the Management of Large Hemispheric Infarction. Neurocrit Care.

[62] Owattanapanich N., Chittawatanarat K., Benyakorn T. (2018). Risks and benefits of hypotensive resuscitation in patients with traumatic hemorrhagic shock: a meta-analysis. Scand J Trauma Resusc Emerg Med.

[63] Marik PE, Kory P, Varon J, Iglesias J, Meduri GU (2021). MATH+ protocol for the treatment of SARS-CoV-2 infection: the scientific rationale. Expert Rev Anti Infect Ther.

[64] Fan E, Brodie D, Slutsky AS (2018). Acute Respiratory Distress Syndrome: Advances in Diagnosis and Treatment. JAMA.

[65] McNicholas BA, Rooney GM, Laffey JG (2018). Lessons to learn from epidemiologic studies in ARDS. Curr Opin Crit Care.

[66] Narendra DK, Hess DR, Sessler CN, Belete HM, Guntupalli KK, Khusid F, Carpati CM, Astiz ME, Raoof S (2017). Update in Management of Severe Hypoxemic Respiratory Failure. Chest.

[67] Ferlini L, Su F, Creteur J, Taccone FS, Gaspard N (2020). Cerebral autoregulation and neurovascular coupling are progressively impaired during septic shock: an experimental study. Intensive Care Med Exp.

[68] Yealy DM, Delbridge TR (1994;24). The shock index: All that glitters. Annals of Emergency Medicine.

[69] Cancio LC, Wade CE, West SA, Holcomb JB (2008;64). Prediction of mortality and of the need for massive transfusion in casualties arriving at combat support hospitals in Iraq. The Journal of Trauma.

[70] Bodo M, Rothwell SW, Dorsey J, Sawyer E, Sipos K (2010). Comparison of circulatory stress indicators in a swine model. Kalokagathia.

[71] Sharma P, Makler V, Chalut C, Rodrigez V, Bodo M (2015). Pyruvate dose-response studies targeting the vital signs following hemorrhagic shock. Journal of Emergencies, Trauma and Shock.

[72] Vargas M, Sutherasan Y, Gregoretti C, Pelosi P (2014). PEEP role in ICU and operating room: from pathophysiology to clinical practice. ScientificWorldJournal.

[73] Kontos HA (1978). Responses of cerebral arteries and arterioles to acute hypotension and hypertension. Am. J. Physiol.

[74] Baumbach GL. (2002). Autoregulation: arterial and intracranial pressure Cerebral Blood Flow and Metabolism 2nd eds: Edvinsson L and Krause DN (Philadelphia, PA: Williams & Wilkins).

[75] Guyton AC (1991). Textbook of Medical Physiology. 8^th^ ed (Philadelphia, PA: Saunders).

[76] Douaud G, Lee S Brain imaging before and after COVID-19 in UK Biobank. MedRxiv.

[77] da Costa L, Fierstra J, Fisher JA, Mikulis DJ, Han JS, Tymianski M (2014). BOLD MRI and early impairment of cerebrovascular reserve after aneurysmal subarachnoid hemorrhage. J Magn Reson Imaging.

[78] Bodo M, Sheppard R, Hall A, Baruch M, Laird M, Tirumala S, Mahon R (2016). Correlation of rheoencephalography and laser Doppler flow: a rat study. Journal of Electrical Bioimpedance.

[79] Greer N, Sayer N, Kramer M, Koeller E, Velasquez T (2016). Prevalence and Epidemiology of Combat Blast Injuries from the Military Cohort 2001-2014.

[80] Kotwal R. S., Montgomery H. R., Miles E. A., Conklin C. C., Hall M. T., McChrystal S. A. (2017). Leadership and a casualty response system for eliminating preventable death. Journal of Trauma and Acute Care Surgery.

[81] Capizzi A, Woo J, Verduzco-Gutierrez M (2020). Traumatic Brain Injury: An Overview of Epidemiology, Pathophysiology, and Medical Management. Med Clin North Am.

[82] Iaccarino C, Carretta A, Nicolosi F, Morselli C (2018). Epidemiology of severe traumatic brain injury. J Neurosurg Sci.

[83] Butler FK, Holcomb JB (2014). Fluid Resuscitation for Hemorrhagic Shock in Tactical Combat Casualty Care: TCCC Guidelines Change 14-01--2 June 2014. J Spec Oper Med.

[84] Jänig C, Forklage R (2021). Triage Decisions in the Context of COVID-19. Old Burden, New Challenge-The Structured Approach for Intensive Care Unit Triage (SAINT) Protocol, Military Medicine.

[85] Hatzfeld JJ, Hildebrandt G (2021). Top 10 Research Priorities for U.S. Military En Route Combat Casualty Care, Military Medicine.

[86] Holcomb JB (2003). Hypotensive resuscitation. Committee on Tactical Combat Casualty Care: Tactical Combat Casualty Care (Washington, DC: Government Printing Agency).

[87] Spinella PC, Holcomb JB (2009). Resuscitation and transfusion principles for traumatic hemorrhagic shock. Blood Rev.

[88] (2010). 121010TCCC Guidelines on Fluid Resuscitation. Recommendations Regarding the Tactical Combat Casualty Care Guidelines on Fluid Resuscitation 201007, Falls Church, VA, Dec 10.

[89] Ahmed A, Bodo M, Armonda RA (2010). Effect of metal fragments in the brain on electrical monitoring: In vitro and in vivo rat studies. J. Phys.: Conf. Ser.

[90] Meghdadi AH, Popovic D, Rupp G, Smith S, Berka C, Verma A (2019). Transcranial Impedance Changes during Sleep: A Rheoencephalography Study. IEEE J Transl Eng Health Med.

[91] Sawell CT, Borsotto M, Reifman J, Hoyt RW (2004). Life sign decision support algorithms. Medinfo.

[92] Bodo M, Pearce FJ, Tsai MC, Garcia A, van Albert S, Armonda R (2013). Cessation of vital signs monitored during lethal hemorrhage: a Swine study. J Spec. Oper. Med.

[93] Eastridge BJ, Mabry RL (2012). Death on the battlefield (20012011): implications for the future of combat casualty care. J Trauma Acute Care Surg.

